# Hepatitis C in North Africa: A Comprehensive Review of Epidemiology, Genotypic Diversity, and Hepatocellular Carcinoma

**DOI:** 10.1155/av/9927410

**Published:** 2025-03-24

**Authors:** Samia Boukaira, Salma Madihi, Hind Bouafi, Zineb Rchiad, Bouchra Belkadi, Abdelouaheb Benani

**Affiliations:** ^1^Department of Molecular Biology, Institut Pasteur du Maroc, Casablanca, Morocco; ^2^Department of Microbiology and Molecular Biology, Faculté des Sciences, Université Mohammed V de Rabat, Rabat, Morocco; ^3^Department of Human Genomics and Genetics, Institut Pasteur du Maroc, Casablanca, Morocco; ^4^Department of Biological Sciences, Faculty of Medical Sciences, Université Mohammed VI Polytechnique EMINES, Benguerir, Morocco

**Keywords:** HCV genotype, HCV prevalence, hepatitis C virus, hepatocellular carcinoma, North Africa

## Abstract

Hepatitis C virus (HCV) is implicated in carcinogenic infections like hepatocellular carcinoma (HCC). Given that no HCV vaccine is currently available, comprehensive epidemiological understanding is crucial for devising effective prevention strategies. In North Africa, existing data on HCV infection and HCV-associated HCC are frequently outdated or limited to specific populations. This systematic review aims to offer new insights into the epidemiology of HCV infection, HCV genotype distribution, and HCV-related HCC in this region. We collected data from electronic databases: PubMed, ScienceDirect, ResearchGate, Google Scholar, and public health reports between 1989 and 2023. We reported the studies carried out in each country in general populations and in groups exposed to HCV infection. Our results show that HCV prevalence varies from 0.5% to 4.61% among the general populations in North African countries. HCV genotype 1 remains the most dominant in the Greater Maghreb region, while genotype 4 is the most dominant in the Nile Valley region. HCC incidence varies between the five countries, and HCV is responsible for 60% of cases, with male dominance. Egypt had the highest number of deaths from HCV-associated HCC. Other factors such as HBV, diabetes, and alcohol use are also responsible for HCC in North Africa. Urban growth and socioeconomic changes have impacted HCV prevalence in the North African region, especially among rural populations, and introduced new risks, such as coinfections and Type 2 diabetes. Here, we propose some recommendations for HCV control and management by patient category in North Africa.

## 1. Introduction

Hepatitis C infection is a transmissible liver disease caused by a small enveloped virus: Hepatitis C virus (HCV) classified in the *Flaviviridae* family and *Hepacivirus* genus [1]. HCV genome exhibits a high degree of variability represented by eight HCV genotypes numbered from 1 to 8, with a multitude of subtypes [2, 3]. HCV infection is often asymptomatic but progresses to chronic infection, cirrhosis, and hepatocellular carcinoma (HCC) in 70% of cases [2]. The WHO estimates that around 58 million people are chronic carriers of the disease, of whom 3.20 million are adolescents and children. The incidence of HCV is around 1.50 million per year, and the mortality rate is around 290,000 per year due to complications like cirrhosis or HCC [2].

HCC is the most common malignant liver tumor, accounting for 90%–95% of all cases, and one of the most common cancers worldwide (5%) [3]. It is the fifth most common cancer in men; the seventh most common in women in terms of incidence, with more than 700,000 new cases diagnosed each year; and the third most common cause of cancer-related death, accounting for 830,000 deaths per year worldwide, or 9.20% of all new cancer cases worldwide (7.90% in men compared with 3.70% in women) [4]. Indeed, 80% of HCC patients are from developing countries [5].

North Africa is made up of five countries, collectively hosting approximately 23% of the African population. It is geographically divided into two subregions: the Maghreb, consisting of Morocco, Algeria, Tunisia, and Libya, and the Nile Valley, represented by Egypt. In this region, updated epidemiological data, including HCV prevalence and incidence, circulating HCV genotypes, sex distribution, age distribution, treatment follow-up, relapses, HCV-related complications, modes of contamination, screening, and HCC situation, are rare.

The purpose of this systematic review is to provide insights into the epidemiology of HCV and HCV-HCC related in North African countries, since the available data in this region are often outdated, or confined to specific small populations, which may be influenced by selection bias.

## 2. Material and Methods

**Figure 1:**
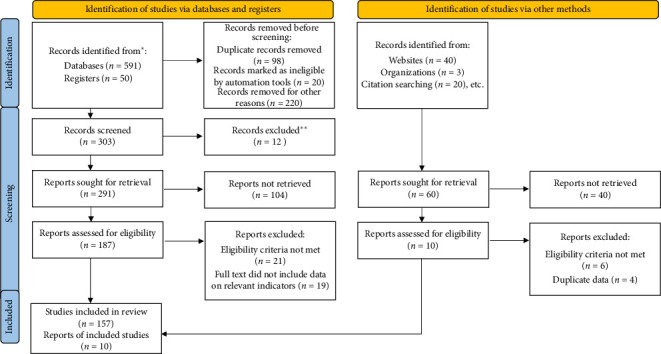
Results of the systematic literature search. ^∗^electronic research, ^∗∗^excluded by automation tools.

We collected and analyzed data on the prevalence of HCV infection, distribution of HCV genotypes, and HCC-HCV associations in North African countries from electronic databases: PubMed, ScienceDirect, ResearchGate, Google Scholar, Web of Science, and Public Health Reports between 1989 and 2024. We included population-based studies that included samples from the following populations: community-based, studies voluntary blood donors, pregnant women, hemodialysis patients, diabetics, thalassemic, inpatients, coinfected patients, healthcare workers, sex workers, drug users, prisoners, and migrants. However, the review primarily focuses on anti-HCV as the biomarker, not HCV RNA. A total of 167 papers were included in this review, according to PRISMA guidelines (Figure 1).

## 3. Results

### 3.1. HCV in North Africa

#### 3.1.1. Morocco

Morocco is a North African country situated in the extreme northwest of Africa, positioned on the border of continental Europe and bordered by both the Atlantic Ocean and the Mediterranean Sea. Morocco has a surface area of 710.850 km^2^ and a population of 36.472 million [6–8], with a Human Development Index (HDI) of 0.683, according to the latest data of 2022, reflecting a medium human development [7].

In this context, Morocco has been actively combating hepatitis C through a national strategic plan since 2016. In fact, several studies have assessed HCV prevalence, with the latest estimate in 2019 at 0.50% [8], lower than the previous 2019 (0.63%), 2017 (0.73%) [9], and 2015–2018 (1.80%) estimates [10]. A large study from 2005–2011 on 41,269 subjects found a 1.58% prevalence among adults, with a higher rate of 3.12% in those over 50 [11]. Earlier, a 2005–2007 study estimated a 1.93% seroprevalence [12].

Among blood donors, the HCV prevalence was 0.62% in a cohort of 169,605 voluntary blood donors conducted in 2013 [11]. A previous study between 1991 and 2010 on a cohort of 19,801 reported a seroprevalence of 0.20% [13], while in another study on 1000 blood donors, the HCV seroprevalence was 1.10% in 1996 [14].

In several studies of chronic hemodialysis patients in Morocco, HCV infection rates varied widely. A 2014 study in Rabat found a 60% prevalence [15]. In Casablanca, a study of 630 patients from 2010–2014 showed a 30.79% prevalence [16], close to a 2013 estimate of 32% [17]. Among 141 patients tested between 2010 and 2012, the prevalence was 12.10% [18]. Another study conducted in Casablanca found a 68.30% prevalence among 303 patients [19]. A higher rate of 76% was reported from a study between 1983 and 2002 on 186 subjects [20]. An earlier study showed a 35.10% prevalence among 114 patients [14].

Moreover, the prevalence of HCV among Moroccans who underwent renal transplants varied from 10.10% to 19.30% in 2010 [21].

Studies on other at-risk populations in Morocco show varied HCV prevalence rates. At Avicenne Rabat University Hospital Center, 2.50% of 601 healthcare workers tested positive [22]. Among intravenous drug users (IDUs), the rate was 60% in 2014 [23], and in 2012, it was 79% in Nador and 46% in Tangier [21]. Regarding women of childbearing age, a 2021 study showed a 0.67% prevalence [24], compared to 1% in pregnant women in 1996 [14]. Among Type 2 diabetics (T2D), the rate was 4.50% in 2014 [25], highlighting the diabetogenic potential of hepatitis C. HCV coinfection in human immunodeficiency virus (HIV)–infected patients was 5.40% [26], 20% among IDUs and sex workers, and 10.6% among diverse socioeconomic groups in 2013 [27]. In 1996, 42.40% of thalassemia patients receiving blood transfusions were HCV positive [28].

HCV prevalence rates among hemophiliacs in Morocco changed across studies. The most recent study showed a 2% prevalence, while a study on children reported 41%. The earliest study found the highest rate at 42%. Among traditional barbers, HCV prevalence dropped from 5% in 2004 to 1.10% in 2011, with a 1.30% rate among their clients [21].

Notably, Morocco, traditionally known as an emigration country, has transformed from a transit nation to Europe but also a destination for a growing number of migrants, reaching 102,400 in 2019. These individuals can serve as a reservoir for various communicable diseases, highlighting the importance of screening. In this context, a cross-sectional study conducted in Oujda on 495 migrants reported an HCV prevalence of 1% [29].

Morocco has made a national strategic plan since 2016 to actively combat hepatitis C. The national prevalence decreased to 0.50% in 2019, although it remains high among certain at-risk groups, including IDUs, hemodialysis patients, and hemophiliacs (up to 42%). Targeted screening and prevention efforts have helped reduce the rates.

#### 3.1.2. Algeria

Algeria has a vast land area of 2.38 million km^2^ and a population of 43.054 million [30, 31]. The country also has a coastline along the Mediterranean Sea. As of today, Algeria's HDI stands at 0.745, indicating a high level of human development [7].

Data on the epidemiology of HCV in Algeria are limited. The latest estimate from 2019 puts the anti-HCV prevalence at 1.74% [32], lower than 2.50% a decade earlier [33]. In El Oued, a northeastern region, the prevalence is 5% [34]. A study of 40 out of 48 regions found high incidence rates in Aïn Temouchent/Sidi Bel Abbes, Algiers, and a significant eastern region, with the eastern region notably having 65% female cases. The most concerning situation was in rural areas in eastern Algeria [35].

Recently, in the province of Bejaia, serological test results over 10 years among blood donors indicated an HCV prevalence of 0.083% [36]. This rate is less than what was found in another study, where the estimated prevalence of HCV was reported as 0.42% [37] and 0.18% documented earlier in 1990 [38].

The only study conducted in Algeria focusing on a high-risk group of hemophiliacs was conducted in 2011 in Batna city and revealed a prevalence rate of 30% [21]. In patients undergoing hemodialysis in Algeria, an HCV prevalence reaching 39% has been estimated [37].

Another group at high risk of HCV is HIV/HCV coinfected people. The seroprevalence in Oran was 1.02%, with transmission routes including dental care (18%), intravenous drug addiction (11%), and other factors [39]. The latter may include unprotected sexual contact, blood transfusions, or organ transplants, and tattooing or body piercing's use of nonsterile needles or equipment during tattooing or body piercing can facilitate HCV transmission.

Results from a cross-sectional study in Algeria indicate a strong association between HCV infection and diabetes, revealing an HCV prevalence of 39.10% among individuals with diabetes mellitus (DM). The prevalence of diabetes was 67.40% in HCV-infected patients with cirrhosis and 33.50% in those without cirrhosis [40].

Estimates of HCV prevalence among pregnant women in Algeria are limited. The few existing data show a prevalence of 2.50% in 2011 [41]. In the Annaba region in 2008, 0.63% of screened pregnant women tested positive for anti-HCV antibodies [42], a higher rate than that reported in an older study carried out between 1992 and 1993 on 715 pregnant women (0.19%) [38].

In Algeria, HCV prevalence decreased to 1.74% in 2019, with higher rates in the east and among at-risk groups, including hemophiliacs, hemodialysis patients, and HIV/HCV coinfected individuals. DM is another important risk factor with a high prevalence among HCV-infected patients in the country.

#### 3.1.3. Tunisia

Tunisia is the smallest among the Maghreb countries with a surface area of 163.610 km^2^ and a population of 11.694 million [6, 7, 33], and is surrounded by the two largest countries in the region, Libya and Algeria. The HDI of the country is 0.731, indicating a high human development [7].

In response to the WHO's global hepatitis program, Tunisia launched a national effort to eliminate hepatitis C in 2016. Studies show that Tunisia has a low HCV prevalence, not exceeding 1% in the general population [21]. However, regional and demographic variations exist. In Central-West Tunisia, the prevalence is 3.32%, higher among women (4.47%) than men (2.16%) [43]. In a comparison of regions, Béja had a 1.70% prevalence, while Tataouine had 0.20%, with the north showing higher rates than the south. Age was the only significant risk factor, with no major differences based on gender or rural/urban residency. A study of young, healthy male adults from 2003 to 2012 reported a stable 0.07% prevalence, decreasing from north to south [44]. An earlier study (1994–1996) estimated the general population's prevalence at 0.71% [45].

Several studies have examined HCV prevalence among Tunisian blood donors. In 2010, a study of 19,783 donors found a seroprevalence of 0.37% [46]. A higher rate (1.18%) was found between 1997 and 1999 in a study on 3480 donors [47]. An earlier study (1994–1997) reported 0.56% [48]. A small sample of 45 donors had 2.20% [49], while a study of 2006 donors revealed 1.09% [50].

A recent nationwide study in Tunisia found a 3.20% prevalence of HCV antibodies among 11,653 hemodialysis patients, with 3.50% in females and 3% in males [51]. Another study reported a 7.30% prevalence among 109 hemodialysis patients [52]. Older studies showed higher rates: 19.07% among 4340 patients in 2003 [53], 20% between 2001 and 2003 [54], 32.60% between 2000 and 2002 [55], and 46.50% in 2000 among 58 patients [56]. Among similar groups, HCV prevalence was 5% in multitransfused patients, 6% in thalassemia patients, and 20.90% in kidney transplant patients [21].

Among local groups of people who inject drugs (PWID) in Tunisia, HCV prevalence rates ranging from 21.70% to 29.10% were recently reported [57], being lower than that reported in 2013 and estimated at 35.80% [58]. Another group, Healthcare personnel, underwent evaluation at Farhat Hached Hospital in the city of Sousse. 885 individuals of 1632 (54%) participated in the study and nine cases tested positive for anti-HCV antibodies, indicating a prevalence of 1% among healthcare workers [59].

Examining HIV/HCV coinfection and HIV infection, a study reported that both were more prevalent in males than females. The primary modes of HIV transmission include heterosexual, homosexual, and drug use, with no significant difference observed between the two gender groups [60].

Groups at intermediate risk were also evaluated for hepatitis C in Tunisia, with the following results: 1% among diabetics, 20% among inpatients and referred patients, a decrease from 40% to 26% among individuals testing positive for HIV, and 1% among female sex workers [21]. Regarding pregnant women, the prevalence of HCV was estimated at 0.50% [37], a higher rate than that reported in 2006 and estimated at 0.20% [61].

In Tunisia, efforts to eliminate HCV began in 2016. The HCV prevalence is below 1% in the general Tunisian population, but higher in specific groups, including hemodialysis and PWID. Some regional and demographic variations (age and gender) in HCV prevalence are reported in the country.

#### 3.1.4. Libya

Situated between Egypt and Tunisia on the southern coast of the Mediterranean, Libya, is the fourth largest country in Africa and the 16th largest in the world, with a surface area of 1.76 million km^2^ and the smallest population in the region at 6.802 million [30, 31, 62]. The country's HDI is 0.718, indicating a high human development [7].

In Libya, HCV prevalence has been well-documented through various studies. Recent data from 368,392 individuals in eastern Libya (Tobruk) show an anti-HCV prevalence of 0.17% [63]. A 2011 study in Tripoli reported a 0.9% prevalence, slightly higher in females, with rates rising from 0.70%–0.90% in those under 30% to 3.7% in those over 30 [64]. A 2008 study of 65,761 individuals found a prevalence of 1.20%, increasing after age 30 [65]. From 1999 to 2001, a study in Tripoli showed a 1.60% prevalence among 800 subjects [66]. Earlier in 1994, a 6.80% prevalence was documented among 266 individuals [67].

Interestingly, in southeast Libya, migrants from sub-Saharan Africa in transit to Europe were recently assessed for viral hepatitis and revealed a positivity for HCV of 31.20%. This group presents a high risk for both the Libyan population and the European populations of the destination, particularly in the case of integration, which can rapidly increase the prevalence of HCV in these countries [68].

The prevalence of HCV in healthy blood donors in Libya was reported to be 1.80% among 1,008,214 participants over a 6-year period (2008–2013) [69]. Earlier in 2002, HCV prevalence was 1.20% [66]. Other studies reported prevalence rates ranging from 0.90% to 6.60% between 1994 and 1995 in the country [67, 70].

Among hemodialysis patients in Libya, a study reported a 16.70% HCV prevalence, with higher rates in the southern regions compared to the western and eastern regions [71]. Over time, HCV prevalence among these patients has decreased. In 2012, a study of 2382 patients from 39 dialysis centers showed a 31.10% prevalence [72], higher than the 20.50% reported between 1991 and 2001 [66]. Older studies in Benghazi revealed prevalence rates of 21% [73] and 42.50% [74].

In other specific groups, the seroprevalence was estimated at 0.36% among pregnant women [41], 2% among healthcare workers [66], 5.20% in women and 7.30% in men among sex workers [75], 8% among 25 cases of acute hepatitis in children in Southern Libya [76], 11% among polytransfused thalassemia patients [77], 22.70% of individuals with a history of blood transfusion [28], 23.70% among prisoners [78], and 94.50% among PWID [79]. Additionally, HCV-positive results were reported among 228 diabetic patients in Libya revealing a prevalence of 10.50%. Specifically, 17% and 8% of Type 1 and T2D were seropositive, respectively [80]. In addition, HIV/HCV prevalence in Tripoli was 0.15%, being the highest in comparison with other coinfections [64].

HCV prevalence varies widely in Libya, from 0.17% to 1.80% among blood donors and 16.70% to 42.50% in hemodialysis patients. High-risk groups include PWID, prisoners, and diabetics. Migrants from sub-Saharan Africa add public health concerns. Interestingly, rates increase with age and in specific populations in the country.

#### 3.1.5. Egypt

Egypt is located in northeast Africa, on the right side of Libya. It is divided into four distinct geographical regions: the Nile Valley and Delta, the Western Desert, the Eastern Desert, and the Sinai Peninsula. The Nile Valley and Delta stands out as a unique feature, not only within Egypt but also across North Africa [81]. The country borders the Red Sea and the Mediterranean and is crossed by the Suez Canal. With a surface area of 1 million km^2^, Egypt is roughly the same size as the American states of Texas and New Mexico combined. It regroups the highest number of inhabitants at 100.39 million [30, 31, 62], and the HDI of the country is 0.731, reflecting a high human development [7].

Egypt has the highest global burden of HCV, not only in North Africa but globally, but has made significant progress in combating the disease [82]. In fact, the WHO recognized Egypt as the first country to achieve “gold tier” status in eliminating hepatitis C, with 87% of HCV cases identified and 93% of diagnosed individuals receiving treatment, surpassing the WHO targets of 80% and 70%, respectively. Recent data show an overall HCV seroprevalence of 4.61% among 48,345,948 people, with the highest rates in Menoufia (8.43%) and the lowest in the Red Sea region (2.17%) [83]. HCV prevalence is higher in men, increases with age, and is more common in rural areas. A 2017 survey across 21 governorates reported a 14.80% seroprevalence, with Menoufia at 37.80%, Beni Suef at 29.20%, and Minya at 28.60% [84]. Earlier, the 2008 Egyptian Demographic Health Survey found a national HCV prevalence of 14.70% [85].

Among blood donors, a 2011 study of 3420 samples from the Alexandria blood bank found that 3.50% were positive for anti-HCV antibodies [86]. Another study conducted in Mansoura on 55,922 first-time volunteer donors reported an 11.95% seroprevalence, declining from 17.70% to 7.40% over the study period [87]. A lower prevalence (2.70%) was found at the same university among student donors in 2006, with higher rates in rural students, a trend still seen in Egypt [88]. Higher HCV prevalence is also noted among paid and family replacement donors compared to voluntary donors [89].

Some populations at intermediate risk of exposure to HCV were reported in Egypt: 2.50% among diabetic children in 2010, a lower level than those reported in previous studies: 44.10% in 2007 and 29.40% in 1995, while among adult diabetic patients the prevalence raised from 20% in 2002 to 60.30% in 2008 [89].

HCV prevalence is higher in certain high-risk groups compared to the general population. Thalassemic patients in Upper Egypt had a prevalence of 37% [90]. Among hemodialysis patients, the prevalence was 7% in 2021 [91], 60.90% in 2016 [92], and 51% in 2015 [93]. IDUs showed a prevalence ranging from 50%–90% in 2013 [89], compared to 63% in 1995 [94]. Healthcare workers had a prevalence of 9% in 2020 [95], down from 17% in 2013 [89]. Pregnant women had a prevalence of 1.70% until 2020, reduced from 8.60% in 2010 [96, 97], and 15.80% in rural Nile Delta villages in 2006. Barbers had a prevalence of 12.30%, and prisoners had 31.40%, according to a meta-analysis [89]. For HIV/HCV coinfections, the prevalence of HIV among HCV patients was 0.64%, predominantly in males [96], while the incidence of HCV among people living with HIV (PLHIV) was 4.06 per 100 person-years in a recent cohort [97].

Egypt has the highest global burden of HCV but has made remarkable progress, achieving WHO “gold tier” status with 87% case identification and 93% treatment rates. HCV seroprevalence dropped to 4.61% nationally, with Menoufia at 8.43%. Higher rates persist in rural areas, hemodialysis patients, and PWID, while prevalence among pregnant women decreased.

HCV prevalence in the general population and among specific groups in the reported North African countries is represented in Table 1.

### 3.2. HCV Genotypes and Subtypes in North Africa

#### 3.2.1. Morocco

The first study on HCV genotype determination in Morocco was carried out in 1997 and indicated that only genotypes 1 and 2 were present among Moroccan patients. The most prevalent subtypes were 1b (47.60%) and 2a/2c (37.10%), with subtype 1a (2.80%) being less common. It was also noted that among hemodialysis patients, only genotype 1 was detected, with a prevalence of 68.40% for subtype 1b and 15.80% for subtype 1a. Subtype 1b was more common among older patients, while subtype 2a/2c was predominantly found in younger ones [98]. In 2011, a study showed that HCV genotypes 1 and 2 were the most common in Morocco, while HCV genotypes 3, 4, and 5 were less frequent. In this study, authors have reported cases of 2i subtype in 93.75% and 2j/2k subtype in 6.25% among 16 cases of 2a/2c and unclassified 2 genotypes [99]. In 2012, a study reported genotypes 1 (46%) and 2 (40%) to be the most frequent, with subsequent representation of genotypes 3 and 4. In IDUs, genotype 1 constituted the majority at 65%, followed by genotype 3 (26%) and genotype 4 (10%) [100]. These results align with those reported in 2014 among IDUs living in the northern region of Morocco where genotype 1a (60.80%) was found in all age groups, followed by genotypes 3a (25.60%) and 1b (4.05%) being present mostly in younger or older ones, respectively, and genotype 4a (9.40%) that remains uncommon, closely mirroring the prevalence in the general Moroccan population [23]. Another examination of HCV subtype distribution among chronically infected patients revealed the prevalence of subtypes 1b (75.20%), 2i (19.10%), and 2k (2.80%), with genotype 4 being exceptionally rare (0.70%). In addition, the prevalence of subtype 1b increased significantly with the severity of the disease from 67.50% in chronic hepatitis C to 84.40% in HCC [100]. Recently, sequencing revealed the presence of new HCV subtypes: 2i in 50%, 1d in 40% of cases, and 2l in 10% among 10 Moroccan chronically infected patients [101]. These reported percentages may differ from one study to another due to sample size and/or type. In this context, we carried out the most recent study in Morocco and the largest one and have reported an HCV subtype distribution of 1b in 37.74%, 2a/2c in 28.18%, 2 in 14.80%, 1 in 9.47%, 1a in 5.14%, 1a/1b in 2.43%, 4 in 0.90%, 3 in 0.57%, 3a in 0.48%, 4a in 0.14%, 5 in 0.10%, and 5a in 0.05%. In this study, advanced liver fibrosis showed an association with genotype 1b, followed by genotype 2a/2c [102].

In Morocco, genotype 1b is the most prevalent HCV subtype, followed by 2a/2c. Among PWID, genotype 1a is most common, with genotype 3a. Genotype 1b is associated with advanced liver fibrosis. New subtypes 2i, 1d, and 2l have also been identified in recent studies (Table 1).

#### 3.2.2. Algeria

In 2013, HCV genotype 1 was reported to be the most frequent (88.70%) in northeastern Algeria, followed by genotypes 2 (8.50%), 4 (1.10%), 3 (0.90%), and 5 (0.20%). Mixed infection across the HCV subtypes was detected in 4.60% of patients. Genotype 1 was significantly less frequent in the ≥ 60 age group than in the younger age group [103]. Inversely, in northwestern Algeria, the genotype 2a/2c was predominant (47%). Another 2013 study conducted in the western region of Algeria revealed that 50% of patients had genotype 2, while genotype 1 was present in 41.47% of cases, with subtype 1b accounting for 71% of those. In contrast, genotypes 3 and 4 were identified in only 1.84% each [104]. In HIV/HCV coinfected patients, the most prevalent genotype was genotype 1 (68.18%), with 1a in 40.91% and 1b in 27.27%. Genotype 2a2c was identified in 22.73% of cases, while genotype 4 was found in a small percentage (9.09%) [39].

In Algeria, HCV genotype 1 was the most common, especially in the northeast, followed by genotype 2. In the northwest, genotype 2a/2c was predominant, with genotype 1, mostly subtype 1b (Table 1).

#### 3.2.3. Tunisia

In Tunisia, studies carried out between 2003 and 2005 reported that genotype 1, particularly subtype 1b, was the most prevalent (80%), followed by genotype 2 (10%). In fact, HCV genotype 2 in Tunisia has received limited research attention; however, a 2014 study revealed that subtype 2c was the predominant circulating subtype (75.50%) [105]. Another study in 2014 demonstrated that subtype 2c was the predominant subtype circulating in Tunisia (65.10%), with co-circulation of isolates from subtypes 2k (11.20%), 2i (5.60%), and 2b (1.10%) [106]. Recently, a 16-year Tunisian retrospective study showed that genotype 1 (subtype 1b) was the most prevalent genotype in the country, accounting for 79.50%, followed by genotype 2 at 13.30%. Genotypes 3, 4, and 5 were detected in 4.80%, 2.20%, and 0.10% of the population, respectively. Mixed infections with different HCV genotypes were identified in 0.10% of the population, with one case each of genotypes 1b + 4, 1b + 2, and 2 + 4. Notably, a significant increase in genotypes 2, 3, and 4 was observed over time. Patients infected with genotypes 1a, 3, and 4 were notably younger than those with genotypes 1b and 2. Additionally, genotypes 1b and 2 were more prevalent in women than in men, while genotypes 1a and 3 were predominantly found in men [107]. In a recent study, subtype 1b was the most prevalent among HCC patients (83.30%) followed by subtypes 1a (3.30%) and 4a (3.30%); meanwhile, one case was untypable [108]. Interestingly, a recent study based on phylogenetic analysis reported new 2 subtypes of HCV genotype 2 in Tunisia: 2v and 2w [106].

In Tunisia, genotype 1, particularly subtype 1b, has been the most prevalent, followed by genotype 2, with subtype 2c being the dominant circulating subtype, and other genotypes (3, 4, and 5) were less common. Genotypes 1b and 2 were more prevalent in women, while genotypes 1a and 3 were more common in men. A recent study identified new subtypes 2v and 2w. Genotype 1b was the most common among HCC patients (Table 1).

#### 3.2.4. Libya

Until 2010, genotypes 1 and 4 were predominant among the patients chronically infected with HCV, 35.70% and 32.60%, respectively [103]. In a cross-sectional study conducted in 2015, the findings showed that genotype 1 was the most prevalent across all regions, ranging from 19.70% to 40.50%, with the highest occurrence in the Tripoli region. Genotype 4 exhibited greater prevalence in the south (49.30%) and west (40%) regions. Genotype 3 had a higher prevalence in Tripoli (21.30%) and the east (15.90%) regions, while genotype 2 was common in the north (23.60%) and south (22.50%) regions. In this study, genotype 1 was the predominant genotype across all age groups, with its highest prevalence observed in individuals below 20 years. It also exhibited the highest prevalence among men at 38.40%. Genotype 2 showed a threefold increase among those aged over 50 compared to the younger population. Genotype 3 was twice as prevalent among individuals aged 20–40 years compared to other age groups. These two genotypes were more common among individuals with a history of IDU abuse. Genotype 4 was most prevalent among women at 39.70%, particularly among those who underwent a cesarean section compared to those not operated [109]. In the same year, a study describing HCV genotypes and subtypes among Libyan patients (Tripoli Medical Center) revealed the prevalence of subtype 1b (14.60%), followed by subtype 4a (5.40%), predominantly among females. Subtype 1a accounted for 4.90%, and subtype 3a was reported in 2.30%. Genotype 2 exhibited heterogeneity, with four reported subtypes: 2a (0.40%), 2b (0.40%), 2c (0.80%), and 2a/2c (1%) [110]. Recently, HCV genotypes 4 (61.50%) and 1 (24.10%) have been identified as the most prevalent among patients, confirming findings from prior studies in Libya. This is followed by genotypes 2 (10.80%) and 3 (3.40%). Notably, genotype 4 was more frequently detected in patients from east Libya (Benghazi) (75.90%) compared to west Libya (Tripoli) (41.60%) and was associated with a higher degree of liver fibrosis. On the other hand, genotype 1 was more prevalent in patients from west Libya (34.10%) compared to east Libya (16.80%) [111], indicating a change in HCV genotype distribution in the country. A recent analysis of HCV genotypes among HIV/HCV coinfected individuals found genotype 1 in 55% of patients, genotype 4 in 25%, and genotype 3 in 20% [112].

Genotypes 1 and 4 were most prevalent in Libya, with genotype 1 being dominant in younger individuals and men. A 2015 study showed genotype 1 was common in Tripoli, while genotype 4 was more prevalent in the south and west. Recently, genotype 4 is the most common, particularly in east Libya, followed by genotype 1. Genotypes 1 and 4 were also dominant in HIV/HCV coinfected individuals (Table 1).

#### 3.2.5. Egypt

HCV genotype 4 is widespread in the Middle East and Africa, with Egypt having the highest prevalence, accounting for almost 90% of infections, followed by genotype 1 that never exceeds 10% [103]. Genotype 4 is a significant contributor to chronic hepatitis, liver cirrhosis, HCC, and the need for liver transplantation in the country. Various subtypes of HCV genotype 4 have been identified in Egypt, including 4a (55%), 4 (24%), 4o (7%), 4m (3%), 4l (3%), and 4n (2%). In 2007, a study even reported an interesting association between subtype 4o and HCC in Egypt [113]. Recently, the findings from a meta-analysis disclosed that genotype 4 constitutes 94.10% of infections in Egypt, with subtype 4a being the most frequently reported (12.10%). Other genotypes have been reported in a minority of cases in the study: genotype 1 (4%), genotype 2 (1.30%), genotype 3 (0.80%), and genotype 5 (0.10%). Genotype 4 was found among blood donors, pregnant women, village residents, outpatients, Egyptian expatriate general populations, thalassemia patients, hemodialysis patients, healthcare workers, children of HCV-infected mothers, household contacts of HCV-infected individuals, hospitalized populations, diabetic patients, HCC patients, chronic liver disease (CLD) patients, acute viral hepatitis patients, special clinical populations, and mixed populations [114].

HCV genotype 4 is predominant in Egypt, accounting for nearly 94.10% of infections, with subtype 4a being the most common. Subtypes such as 4m, 4l, 4o, and 4n identified, with subtype 4o linked to HCC. Genotype 4 is widespread across various populations, including blood donors, pregnant women, patients with CLD, and healthcare workers (Table 1).

### 3.3. Cirrhosis in North Africa

In the North African region, HCV infection is highly prevalent in cirrhosis cases, with 83% of cirrhosis cases attributed to HCV [115].

#### 3.3.1. Morocco

In a study of 699 patients with chronic hepatitis C, the overall prevalence of cirrhosis (stage F4) was estimated at 31.80%. This cirrhosis is distributed as follows: F4.1 (cirrhosis without varices or severe events) in 14.60% of patients, F4.2 (cirrhosis with varices, without severe events) in 13.90%, and F4.3 (cirrhosis with severe events) in 3.30% [116].

A more recent study involving 150 patients analyzed the relationship between the genotype of HCV and the progression of liver fibrosis. Genotype 1b, the most prevalent in Morocco, was strongly associated with advanced stages of fibrosis: 26.92% of patients with this genotype were at Stage F3 (advanced fibrosis), and 68.85% were at Stage F4 (cirrhosis). Genotypes 2a/2c, although less frequently associated with advanced stages of fibrosis, also significantly contribute to disease progression, with a prevalence of 18.03% at Stage F4 (cirrhosis) [102].

These results highlight the importance of identifying the HCV genotype to assess the risk of progression to cirrhosis and to tailor management strategies accordingly.

#### 3.3.2. Algeria

A study on 337 Algerian patients with HCC found that 81.50% developed the tumor on cirrhotic livers. The mean age was 63.80 years, with a male-to-female ratio of 1.5. Key risk factors included hepatitis C (48.70%), chronic hepatitis B (21.80%), and metabolic comorbidities such as overweight (45%) and diabetes (32.80%). The study highlights regional disparities in patient distribution, with higher proportions in Algiers, Batna, and Tipaza [117].

Another study conducted at the University Hospital of Oran assessed the effectiveness of the MELD-Na score in predicting three-month mortality among cirrhotic patients. Of 47 patients, 17% died, and the MELD-Na showed slightly better predictive accuracy (AUC: 0.952) compared to the classic MELD (AUC: 0.931). The optimal threshold for MELD-Na was 21, with a sensitivity of 87.5% and specificity of 87.20%. The causes of cirrhosis were mainly viral and metabolic, including 23% hepatitis C, 19% nonalcoholic steatohepatitis (NASH), 13% autoimmune hepatitis, 6% alcohol consumption, and 4% hepatitis B. These findings highlight the significant prevalence of viral and metabolic causes of cirrhosis [118].

#### 3.3.3. Tunisia

A study explored the impact of genetic polymorphisms (IL-18, IFN-c, and IL-10) on the risk of cirrhosis in chronic hepatitis C patients. Among 77 chronic HCV carriers, 31 (40%) had cirrhosis. The study highlights the crucial role of genetic factors in the progression of hepatitis C to cirrhosis [119].

In another study, the cohort included 77 patients, 31 of whom had cirrhosis (40%), primarily caused by hepatitis C (48.70%) and B (21.80%), as well as cryptogenic cases. The median age of the patients was 64 years [120].

Among 53 chronic hepatitis C patients, 30% had cirrhosis, 41.50% had moderate fibrosis (F1/F2), and 58.5% had severe fibrosis (F3/F4). The median age was 53.1 years, with mean AST of 82.5 IU/L and ALT of 104 IU/L [121].

#### 3.3.4. Libya

A total of 286 patients with chronic HCV infection from the two main tertiary care hospitals in Benghazi and Tripoli were studied, including 138 males and 148 females. The mean age was 45.0 ± 13.8 years. The majority of patients (69.90%) had fibrosis stage 2–4. Stage 0 or 1 fibrosis was observed in 30% of patients [111].

#### 3.3.5. Egypt

An Egyptian systematic review included 13 studies on patients with compensated HCV cirrhosis, evaluating outcomes like death, transplantation, and liver decompensation. The annual risk of death/transplantation ranged from 2.74% to 6.72%, and HCC risk from 1.51% to 7.14%. Risk factors for cirrhosis progression included low albumin, high bilirubin, platelet count, age, and presence of esophageal varices [122].

Another study involved 640 chronic hepatitis C patients in Egypt, with a mean age of 49.89 years. The majority had a history of blood transfusion, surgery, and schistosomiasis. Liver cirrhosis was diagnosed in 46.90% of patients. After follow-up, 10% of patients developed HCC [123].

A larger study analyzed 1013 CLD patients in Egypt reported that the majority had viral etiology (83.5%), with HCV being the dominant cause (80.30%), whereas HBV and schistosomal etiology affected 3.20% and 11.1%, respectively. 5.4% had unidentified causes. About 50% presented with advanced cirrhosis, with ascites (39.20%) and gastrointestinal bleeding (22.80%) being common. HCC was observed in 9.30%. T2D was prevalent in 22.80% of the patients, mostly in those with viral etiology. HCV remains the leading cause of CLD in the Nile Delta region [124].

### 3.4. HCC in North Africa

Around 85% of HCC cases occur in developing countries, where hepatitis C infection is a major risk factor [5, 36]. It is a significant contributor to HCC, leading to 905,700 new cases diagnosed each year [125], worldwide. In Africa, liver cancer cases contribute to 7.80% of the global incidence and 8.1% of global mortality [126]. In North Africa, a multicenter study of the risk factors for HCC was carried out in cooperation between Morocco, Algeria, and Tunisia and showed that HCV was responsible for 60% of HCC in this region. The mean age of patients diagnosed with HCC was 62 ± 10 years [127].

#### 3.4.1. Morocco

In Morocco, the annual incidence of HCC is 3.20/100,000 individuals, with liver cancer-related mortality reaching 3.10/100,000 individuals, according to 2020s data [128]. This reflects an increase from the 1990 mortality rate of 1.73/100,000 individuals [129]. In a recent analysis, 148 cases HCC in cirrhotic livers were examined, revealing a predominance of females with a sex ratio of 1.24. The study indicated that the etiology of cirrhosis was primarily linked to HCV (53.37%) and HBV (26.35%) [130]. Inversely, a study covering the period from 2001 to 2015 and involving 440 cases of HCC, indicated a higher prevalence of liver cancer in males (62.50%) compared to females (37.50%), with HCV identified as the primary etiological factor [131]. Other studies reported that HCV was the leading cause (36%), followed by HBV (31%), alcohol consumption (14%) and other causes (19%) [121, 122]. Earlier in 2009, a study on 96 HCC cases reported that the prevalence of HCV among patients with HCC was 57% [132]. In this context, it is worth noting that changes in Moroccan epidemiology, such as the rise in obesity and diabetes incidence along with significant shifts in dietary habits, may impact the epidemiology of HCC in the country. These changes should be considered in the development of national health programs [133] (Table 2).

#### 3.4.2. Algeria

Up to 2012, the annual incidence of HCC in Algeria ranged from 4 to 7.90/100,000 individuals [134]. By 2017, the reported incidence had decreased to 2/100,000 individuals-year, with an associated mortality rate of 2.14/100,000 individuals-year [129]. As per the most recent WHO data released in 2020, the HCC incidence was 1.50/100,000-year and the liver cancer accounted for 551 deaths in Algeria, representing 0.30% of total deaths and the age-adjusted death rate was reported as 1.40/100,000 of the population [118, 126]. In a recent investigation involving 337 cases of primary liver cancer in Algiers, the male-to-female sex ratio was 1.5. The most prevalent risk factors identified were HCV, HBV, and metabolic pathologies, including T2D and obesity. The study highlighted a notable duality in risk factors and tumor presentation between male and female patients. Women tended to be older and exhibited higher seropositivity for anti-HCV (60%) [117]. In another study conducted from October 2014 to October 2018 in Oran on 19 patients with HCC, the findings revealed a male predominance of 73.60%. Identified risk factors included HCV in 36.84%, smoking in 25%, alcohol use in 14%, diabetes in 17.80%, and HBV in 10% [135] (Table 2).

#### 3.4.3. Tunisia

In Tunisia, there have been significant shifts in the contribution of viral risk factors to liver cancer over the last few decades. Until the early 2000s, HBV was the predominant risk factor, with estimated prevalence of HBsAg and HCV in HCC patients at 60% and 19%, respectively. Since 2010, there has been a decline in HBsAg prevalence to around 25%, while the prevalence of anti-HCV has varied between 47% and 62% [108]. In 2018, the incidence of HCC was reported to be 1.49/100,000 cases annually in Tunisia, with men more affected with a prevalence of 90.33% and HCV being the leading cause of HCC (44%), followed by HBV (20%), alcohol consumption (18%), and other etiologies (18%) [122, 129]. Recently in 2020, the HCC incidence was estimated at 4.30/100,000 annually and the mortality rate due to liver cancer in the country was estimated at 4 cases per 100,000 individuals-year [128]. In a recent study involving 73 Tunisian patients with HCC, it was observed that liver cancer occurred in cirrhotic livers in 72% of cases, with a male predominance (sex ratio M:F of 1.1). HCV infection was the predominant risk factor, accounting for 64.30%, thereby affirming findings from earlier studies. Notably, patients hailing from Western Tunisia, which shares a border with Algeria, constituted 64% of cases, primarily composed of women. This subgroup displayed significantly higher occurrences of tattoos or scarifications (83%) and HCV infection (80%) compared to individuals from other regions of the country [108] (Table 2).

#### 3.4.4. Libya

Between 2003 and 2005, HCC accounted for about 3% of all cancers according to the Benghazi cancer registry in Libya. Its incidence was estimated at 8 to 11.90/100,000 cases per year in 2012 and HCV was the leading cause of HCC in Libya (34%), followed by HBV (33%), alcohol (15%), and other causes (18%) [136]. In 2020, the HCC incidence decreased in the country to 4.40/100,000 and the mortality to 4.20 cases per 100,000 [128] (Table 2).

#### 3.4.5. Egypt

In Egypt, HCC poses a considerable public health challenge. In fact, it is the fourth most common cancer in the country [137], accounting for a disproportionately high percentage of cancers, particularly in men, where it comprises 33.63% of all cancer cases, compared to 13.54% in women [138]. In addition, Egypt is the second country globally after Mongolia affected by HCC with an incidence of 34.10/100,000 cases annually [139], and men are the most affected with an incidence of 2 to 3 times higher than females and a prevalence of 81%. HCC is the leading cause of cancer-related mortality and morbidity in Egypt. In fact, in 2020, HCC was responsible for more than 26,000 deaths in the country [140], with a mortality's age-standardized rate of 32.50/100,000 [128]. This type of liver cancer is associated with various predisposing conditions, including HCV (63%), HBV (13%), alcohol consumption (12%), and other causes (12%), as well as both alcoholic and nonalcoholic cirrhosis [122, 125]. Additionally, DM has been identified as a contributing factor in 30% of Egyptian HCC patients, highlighting the importance of metabolic conditions in the pathogenesis of liver cancer. Given these contributing factors, the growing prevalence of HCV and diabetes, as well as the high rates of cirrhosis, underlines the urgency for enhanced screening, early detection, and effective management strategies for HCC in Egypt [138] (Table 2).

## 4. Discussion

Understanding the evolving epidemiology of viral hepatitis-related HCC in Africa is crucial for identifying key intervention areas to prevent it. HCV-related HCC in North Africa serves as a significant example, as the Middle East and North Africa (MENA) region is recognized as the most heavily impacted area globally by HCV infection.

In this review, we have reported the decrease in HCV prevalence among the general population over time in all North African countries. The current prevalence varies from low to intermediate, similar to that in developed nations, hovering around 1%, except for Egypt, being the most affected country, due to extensive campaigns involving parenteral antischistosomiasis treatment and unreliable sterilization practices conducted in the country during the 1960–1970 period [27].

Gender analysis revealed no significant difference in HCV infection rates in Morocco, while in Algeria and Tunisia, women were more affected by the virus. Conversely, studies in Libya and Egypt indicated a predominance of men. The distribution is also influenced by age and regional factors. Contributing factors to the HCV epidemic, especially in rural areas, include interfamily transmission, work-related accidents among healthcare workers, and the use of unsterilized equipment in tattooing, piercing, dental care, and barbering. Due to the diversity within regions and populations, prevalence classifications remain imprecise.

Despite efforts to control HCV transmission, certain groups, particularly PWID, show significantly higher prevalence rates compared to the general population. HCV prevalence among PWID is alarmingly high, reaching 60% in Morocco, 94.50% in Libya, and 50%–90% in Egypt, well above the MENA region's average of 31.70%. While IDU is a major contributor to HCV transmission in low-prevalence, high-income countries, in low- and middle-income countries, unsafe medical practices and community behaviors play a larger role. Reducing needle-sharing among PWID could lead to a 16% reduction in HCV infections in the MENA region and 43% globally by 2030 [141]. Second, hemodialysis patients in North Africa are highly affected by HCV due to nosocomial transmission linked to dialysis duration, blood transfusions, and shared syringes, with HCV RNA being resistant to room temperature for up to 48 h. Third, the prevalence of anti-HCV in blood donors was high compared to the general population in Morocco, Libya, and Egypt. Interestingly, we noted a decrease in HCV prevalence over time in Morocco, Tunisia, and Egypt, in particular, due to the increase in preventive and safety measures in blood centers to guarantee safety and reduce the risk of post-transfusion infections. Fourth, HCV prevalence among pregnant women ranges from 0.36% to 2.50%, and a 2019 global study found approximately 14.86 million women of childbearing age affected by HCV, with the highest prevalence in the Eastern Mediterranean region at 1.75%, increasing with age [24]. Fifth, the prevalence of HCV among healthcare workers ranges from 1% to 9%, with very few instances of transmission from healthcare providers to patients. As a result, most healthcare personnel infected with HCV may not need to modify their professional duties based on their infection status [142].

On the other hand, studies have indicated that over 33% of individuals with chronic HCV infection are likely to experience at least one extrahepatic manifestation, including diabetes [143]. In the present review, HCV prevalence rates ranged from 1% to 60.30% in North African countries and were higher in adult diabetics than in children. Indeed, persistent HCV infection has the potential to increase the risk of diabetes onset, T2D in particular. This is attributed to the virus's influence on the liver, a key organ in glucose storage. If the liver fails to function optimally, it may result in elevated blood glucose levels and insulin resistance. Furthermore, infection with hepatitis C, particularly its treatment involving interferon α (IFNα), has the potential to initiate the onset of Type 1 diabetes [143].

Coinfection rates of HCV and HIV in North Africa range from 0.15% to 26%, with Libya having the lowest and Tunisia the highest. In Egypt, the high HCV incidence among PLHIV (4.06/100 person-years) aligns with reports of the fastest growing HIV epidemic in the MENA region. This is largely due to the lack of effective prevention programs, such as opioid substitution therapy, needle exchange programs, and inadequate HCV screening in high-risk groups like men who have sex with men (MSM), although screening for PWID is in place to meet WHO's HCV elimination targets [97]. Conversely, Morocco exhibits a higher rate of HIV among individuals with HCV infection, yet it stands as the only country in the North African region equipped with reliable surveillance programs for HIV [27].

In addition, it has been shown that immigrants from North Africa to Europe have a higher prevalence than the general population of the European Union in the host countries, which raises the interest of national screening programs for immigrants and the administration of treatment [136, 137, 144].

Few studies have focused on specific categories at high risk of HCV such as hemophiliacs, organ donors/recipients, prisoners, sex workers, barbers, and their clients and people who follow traditional medical practices, with circumcision as a major example. In developing nations, many barbers are often illiterate and lack awareness of health-related infectious agents, contributing to the spread of infections through the reuse of unsterilized razors and scissors [145], thus recognizing that traditional barbers and their clients could face an elevated risk of HCV that provides valuable insights from a public health perspective. Over time, many of these traditions have changed or even disappeared.

HCV genotype 1, particularly subtype 1b, is predominant in Morocco, Algeria, Tunisia, and Libya, accounting for over 50% of infections, with subtype 1a also common. Morocco uniquely reports subtype 1d. Genotype 2 is notable in Algeria and Morocco, with subtype 2a/2c being most prevalent, and Tunisia reporting several subtypes, including 2v and 2w, which are newly identified. Egypt is dominated by genotype 4 (> 94%), with subtypes 4a, 4l, 4o, 4m, and 4n. Genotype 4 is also present in Libya, but rare in Morocco. Genotypes 5–8 are limited in the region, and the prevalence of HCV genotypes varies by region and transmission mode, with injection drug use linked to genotypes 1a and 3a, and blood transfusion to genotypes 1b and 2 [146]. Genotype 1b exhibited a tendency for increased virulence [102].

In North Africa, the number of HCC cases is multiplied by 10 in the case of HBV infection and by 33 in the case of HCV infection [127]. In terms of incidence, we reported the rates of HCC to vary from 1.50 to 34.10/100,000 cases annually across North African countries, with Algeria exhibiting the lowest rate and Egypt recording the highest one. Egypt also experienced the most substantial increase in the age-standardized incidence rate (ASIR) for HCV-related liver cancer from 1990 to 2017 in this region, with a notable rise of +47.1% [129], and high rates being reported in rural and urban areas [113]. The incidence of HCC remains high even in developed countries. According to Globocan 2022, the age-standardized rate in France is 7.80 [147], even though France is also in the intermediate HCV prevalence zone [2]. In France, the HCC incidence is majority of males (88%) and only 12% of females, while 84.49% of the etiology is due to alcohol consumption, compared with 15.51% for HCV [148, 149]. The present review highlights other etiologies of HCC in North Africa, including HBV, diabetes, alcohol consumption, and tattoos. Egypt has the highest number of deaths due to HCV-associated HCC, followed by Libya, Tunisia, and Morocco. The incidence and mortality of HCC are expected to rise in African nations, driven by ongoing challenges in managing endemic viral infections and environmental risk factors, compounded by resource limitations [148].

Analysis by gender has showed a male predominance, possibly attributed to the conservative nature of the countries. This suggests that certain modes of transmission and aggravating factors, such as smoking, are more prevalent among men than women, particularly in Libya [149]. Inversely, the latest studies in Morocco and Western Tunisia show a predominance of women with regard to HCC. The liver's morphological and functional characteristics are influenced by sex hormones, as it expresses receptors for both estrogen and androgen. Both androgens and estrogens can stimulate hepatocyte proliferation, potentially acting as inducers or promoters of liver tumors. Additionally, prolonged use of oral contraceptives and anabolic androgenic steroids may contribute to the development of benign liver tumors (such as hemangiomas, adenomas, and focal nodular hyperplasia) as well as malignant HCC [150]. Studies indicate that estrogens have an anti-inflammatory effect, improving outcomes in severe infections, wound healing, and repair [151], explaining the overall gender difference in regard to HCC.

Finally, the population of North Africa exhibits Mediterranean characteristics rather than African ones in terms of race, customs, and cultural background. In fact, the main factor contributing to the development of HCC in black South Africans is HBV infection, with HCV infection following as a secondary risk factor. Similarly, HBV is the primary contributor to HCC in developing nations, whereas HCV is the main cause in developed countries [152]. Other than that, HCC tends to manifest at a younger age in Africa compared to other regions globally, typically leading to a poor prognosis and resulting in the loss of many individuals during their most productive years [126]. Additionally, from a development point of view and socioeconomic changes, urbanization has contributed to a general improvement in health in the North African region [153] and several studies have shown that the level of urbanization may influence HCV prevalence in the North African region, reporting higher prevalence among rural populations [46, 141, 154]. Moreover, the quick rise in urbanization has led to a significant increase in additional risk factors such as coinfections and Type 2 DM, being the most common form of diabetes (90%–95%) in North African countries and sub-Saharan Africa, and including obesity, physical inactivity, aging, and nutrition transitions [148].

## 5. Conclusion

HCV infection poses a significant global public health challenge, particularly in North Africa. It is a leading cause of HCC in the region, alongside other risk factors such as HBV infection, diabetes, alcohol consumption, and tattoos. Additionally, rapid urbanization and socioeconomic changes have introduced new risks, including coinfections and T2D. In the absence of an effective hepatitis C vaccine, early detection and treatment are essential to control the spread of the infection and mitigate its complications.

Recommendations for HCV control by patient category:

• PWID: Given their significant role in HCV transmission, PWID are often marginalized. It is vital to implement targeted public health initiatives in North Africa and beyond to address their specific challenges and needs.
• Hemodialysis Patients: Identifying the prevalence of HCV infection among this category is critical for developing strategies to prevent transmission within dialysis settings, including the adoption of stringent infection control measures.
• Pregnant women: Although HCV rates are low among pregnant women in North Africa, routine screening during prenatal care is essential. Early detection and treatment can prevent mother-to-child transmission, which is estimated at 4%–8% in HIV-uninfected mothers and 17%–25% in those coinfected with HIV.
• Traditional barbers: Identifying HCV risk factors associated with traditional barber practices can enhance our understanding and help develop strategies to reduce HCV transmission within the population.
• Healthcare workers: This sensitive category should adhere to standard precautions, which may involve the proper utilization of personal protective equipment such as gloves, masks, and protective eyewear, depending on the specific medical procedure.

Further predictive studies in the North African context are urgently needed to deepen our understanding and improve the management of HCV-related issues in the region.

## Figures and Tables

**Table 1:** HCV prevalence and genotypes and subtypes in North Africa.

Country	Morocco	Algeria	Tunisia	Libya	Egypt
HCV prevalence (%)					
General population	0.50	1.74	< 1	1.20	4.61
Blood donors	0.62	0.42	0.37	1.80	11.95
Hemodialysis	60	39	3.20	16.70	7
IDUs	60	—	21.70–29.10	94.50	50–90
Pregnant women	1	2.50	0.50	0.36	1.70
Other					
HIV (+) patients	5.40–20	1.02	26	0.15	0.64
Diabetics	4.50	39.10	1	10.50	2.50–60.30
Healthcare workers	2.50	—	1	2	9
Thalassemics	42.40	—	6	11	37
Hemophiliacs	2	30	—	—	—
Kidney transplants	10.10–19.30	—	20.90	—	—
Barbers	1.10	—	—	—	12.50
Prisoners	—	—	—	23.70	31.40
Sex workers	—	—	1	5.20–7.30	—
Migrants	1	—	—	31.20	—
Multitransfused	—	—	5	22.7	—
Inpatients and referred patients	—	—	20	—	—
Acute hepatitis	—	—	—	8	—
HCV genotypes/subtype prevalence (%)					
1	9.47–65	41.47–88.70	—	16.80–55	4–10
1a	2.80–60.80	40.91	—	4.90	—
1b	4.05–75.20	27.27–71	79.50–80	14.60	—
1a/1b	2.43	—	—	—	—
1d	40		—	—	—
2	14.80–40	8.50–50	10–13.30	10.80–23.60	1.30
2a	—	—	—	0.40	—
2a/2c	28.18–37.10	22.73–47	—	1	—
2b	—	—	1.10	0.40	—
2c	—	—	65.10–75.50	0.80	—
2i	19.10–93.75	—	5.60	—	—
2k	2.80	—	11.20	—	—
2l	10	—	—	—	—
2j/2k	6.25	—	—	—	—
2v	—	—	ND	—	—
2w	—	—	ND	—	—
3	0.57–26	0.90–1.84	4.80	3.40–21.30	0.80
3a	0.48–25.60	—	—	2.30	—
4	0.70–10	1.10–9.09	2.20	25–75.90	24–94.10
4a	0.14–9.40	—	—	—	12.10–55
4c/4d	—	—	—	2.30	—
4l	—	—	—	—	3
4o	—	—	—	—	7
4m	—	—	—	—	3
4n					2
5	0.10	0.20	0.10	5.40	0.10
5a	0.05	—	—	—	—
Mixed	—	4.60	0.10	—	—

Abbreviation: ND, newly described.

**Table 2:** Incidence, mortality, and etiologies of HCC in North African countries.

Country	Incidence (cases/100,000)	Mortality (cases/100,000)	Dominance	Etiologies (%)			
				HCV	HBV	Alcohol	Other
Morocco	3.20	3.10	Female (F:M: 1.24)	36–57	26.35–31	14	ND: 19

Algeria	1.50	1.40	Male (M:F: 1.50)	36.84	10	14	Diabetes: 17.8

Tunisia	4.30	4	Male (M:F: 1.10)	44–80	20	18	ND: 18
							Tattoos/scarifications: 83

Libya	4.40	4.20	—	34	33	15	ND: 18

Egypt	34.10	32.50	Male (81%)	63	13	12	ND: 12
							DM: 30

Abbreviations: DM, diabetes mellitus; F, female; HBV, hepatitis B virus; HCV, hepatitis C virus; M, male; ND, not determined.

## Data Availability

The epidemiological data supporting this systematic review are from previously reported studies and datasets, which have been cited. The processed data are available from the corresponding author upon request.
